# Self-Administered Cognitive Rehabilitation Using an Electronic Device in Subacute Stroke Patients: A Proof-of-Concept Study on Safety, Feasibility, and Preliminary Efficacy

**DOI:** 10.3390/neurosci6040109

**Published:** 2025-10-30

**Authors:** Cristina Fonte, Alessio Damora, Laura Abbruzzese, Giorgia Rotundo, Alessandro Picelli, Ylenia Gallinaro, Elisa Evangelista, Mauro Mancuso, Nicola Smania, Valentina Varalta

**Affiliations:** 1 Neuromotor and Cognitive Rehabilitation Research Centre (CRRNC), Section of Physical and Rehabilitation Medicine, Department of Neuroscience, Biomedicine and Movement Sciences, University of Verona, P.le L.A. Scuro 10, 37134 Verona, Italy; cristina.fonte@univr.it (C.F.); giorgia.rotundo@univr.it (G.R.); alessandro.picelli@univr.it (A.P.); yleniagallinaro@gmail.com (Y.G.); elisa.evangelista@univr.it (E.E.); nicola.smania@univr.it (N.S.); valentina.varalta@univr.it (V.V.); 2 CRT Tuscany Rehabilitation Clinic, Piazza del Volontariato 1, 52025 Montevarchi, Italy; alessio.damora@gmail.com (A.D.); laura.abbruzzese@libero.it (L.A.); 3 Neurorehabilitation Unit, Department of Neurosciences, Hospital Trust of Verona, 37134 Verona, Italy; 4 Functional Recovery and Rehabilitation Unit National Health Service, 58100 Grosseto, Italy

**Keywords:** stroke rehabilitation, telemedicine, therapy, computer-assisted

## Abstract

Background: Cognitive impairment after stroke often reduces independence and quality of life. Cognitive rehabilitation is therefore essential, and recent research on computer-based interventions has shown promising results. This proof-of-concept study investigated the effects of additional self-administered cognitive training using an electronic device, compared with traditional paper-and-pencil methods, on attentional functions in individuals with subacute stroke. Methods: Participants were randomly assigned to an experimental group or a control group. For two consecutive weeks, both groups received forty-five-minute, face-to-face cognitive therapy sessions each morning, delivered via an electronic device. In addition, the experimental group engaged in sixty minutes of self-administered cognitive training using the same device, while the control group completed conventional exercises with paper-and-pencil tools. Neuropsychological assessments were conducted before and after the intervention. Results: Twenty-three participants were included (experimental group: eleven; control group: twelve). No significant differences in safety or attentional performance were observed between groups. Within-group analyses showed improvements in the experimental group in attentional shifting, inhibitory control, visuospatial planning, and problem-solving, while the control group improved in visuospatial planning and problem-solving. Conclusions: These preliminary findings suggest that self-administered electronic cognitive training may be a feasible approach to support attentional recovery in individuals with subacute stroke.

## 1. Introduction

Stroke is a leading cause of long-term disability in adults and is frequently associated with functional, motor, and cognitive impairments. A substantial proportion of survivors experience deficits in attention, memory, spatial awareness, and executive functioning, which limit independence in Activities of Daily Living and reduce Quality of Life [1,2]. Clinical strategies therefore aim to initiate functional recovery as early as possible [3,4].

Attentional deficits are among the most common cognitive impairments post-stroke, affecting 46–92% of patients in the acute phase [5,6], and 24–51% at discharge [7]. Information processing speed is also frequently compromised, with estimates of 50–70% [8,9]. Although some recovery may occur [10], 20–50% of individuals experience persistent difficulties for years [11,12]. For example, Turunen et al. reported that 34% of patients in the early weeks post-stroke had processing speed deficits, with partial recovery in six months and minimal gains thereafter [6]. Middleton et al. found psychomotor processing speed to be among the most impaired functions, affecting 53.5% in the subacute and 33.7% in the chronic phase [13]. These deficits manifest as reduced concentration, distractibility, difficulties in error monitoring, multitasking problems, cognitive slowing, and mental fatigue [14,15,16], impacting complex domains such as language, memory, planning, and problem-solving [17], and are linked to poorer motor recovery, greater dependence, and increased supervision needs [18].

In recent years, technology-based interventions, particularly computer-delivered programs, have shown promise in cognitive rehabilitation. Delivered via electronic devices, they use multimedia resources to target memory, attention, perception, and spatial neglect [19,20,21,22,23,24]. De Luca et al. [1] found greater cognitive gains, including in memory, attention, spatial cognition, and executive functioning compared to conventional paper-and-pencil exercises. Clinical trials have confirmed benefits in restoring cognitive abilities and reducing recovery time [21,22,23,24,25,26,27,28,29], with additional evidence in neurodegenerative conditions [29], multiple sclerosis [25,26], and Parkinson’s disease [27]. Advantages of device-based rehabilitation include optimization of treatment duration, reduced clinician workload, and feasibility for telerehabilitation [30]. These tools deliver individualized, repetitive activities with immediate feedback, promoting autonomy, progress tracking, and adherence [22], and enabling continuity of care after discharge [16]. Their adaptability enhances engagement and efficacy [31,32], complements traditional methods, and allows precise control of stimuli and task execution [33,34].

Given these benefits, standardized, accessible, and user-friendly protocols are needed to ensure independent operation and maximize outcomes. This proof-of-concept study aimed to preliminarily evaluate whether additional self-administered cognitive training using an electronic device is a feasible, safe, and potentially effective approach for individuals with subacute stroke, compared with additional self-administered paper-and-pencil exercises.

## 2. Materials and Methods

### 2.1. Trial Design

This proof-of-concept study employed a single-blind, randomized controlled design. The examiner responsible for outcome assessments was blinded to group allocation, which was determined using a 1:1 randomization ratio. All participants provided written informed consent prior to enrollment. The research was conducted in accordance with the principles of the Declaration of Helsinki and was approved by the Ethics Committee of Neuroscience, Biomedicine, and Movement Sciences Department at the University of Verona (Protocol CE/269666; 2 October 2017). The study is reported in accordance with CONSORT guidelines and is registered on ClinicalTrials.gov (Identifier: NCT06755437).

### 2.2. Participants

Patients were recruited between October 2017 and December 2021 from the Neuromotor and Cognitive Rehabilitation Research Center of the University of Verona. Inclusion criteria were ischemic or hemorrhagic stroke within two weeks of onset; age under 85 years; and the presence of attentional deficits as measured by the Trails Task and/or the Broken Hearts subtest of the Oxford Cognitive Screen (OCS) [35]. Exclusion criteria were documented visual impairments in clinical history (e.g., cataracts, diabetic retinopathy, glaucoma, hemianopia); psychiatric disorders not pharmacologically stabilized; drug or alcohol abuse; history of dementia; severe comprehension deficits as determined by the Semantics subtest of the OCS [35]; and spatial neglect as assessed by the Broken Hearts subtest of the OCS [35]. Eligibility assessment was carried out in collaboration with the physiatrists at our Center.

### 2.3. Blinding and Allocation

Participants were randomly assigned to one of two groups: the experimental group or the control group, following a 1:1 allocation ratio. Group assignment was conducted by an external investigator (V.V.) using a balanced (restricted) software-generated randomization scheme. A separate investigator (C.F. or A.D.), blinded to group allocation, performed the neuropsychological evaluations before (T0) and after the intervention (T1).

### 2.4. Treatment Procedures

**Figure 1:**
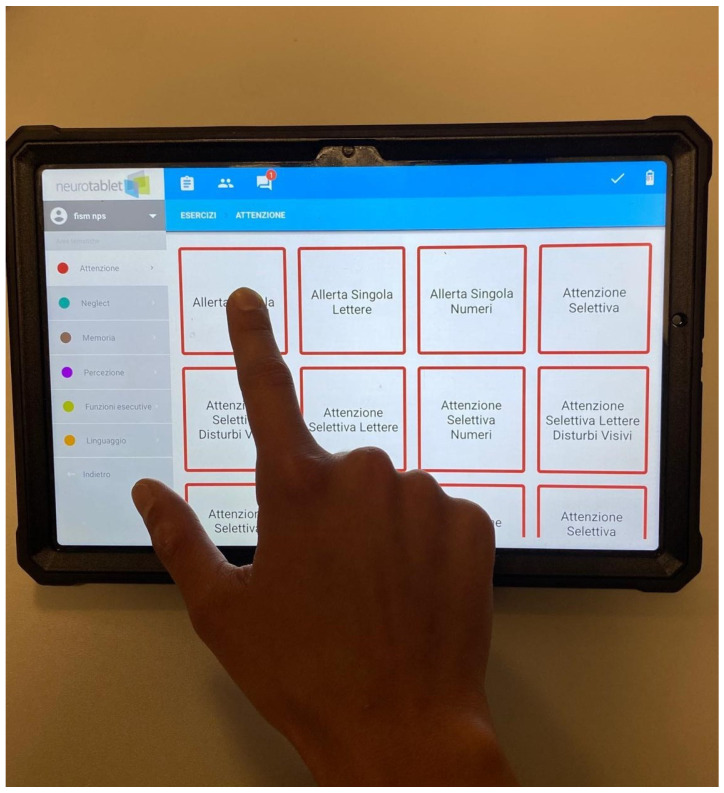
The Neurotablet^®^ device consists of a Samsung Galaxy tablet with its platform preinstalled. Above is the initial interface of the Neurotablet^®^ platform.

Each day, both groups received the same forty-five-minute face-to-face cognitive training using an electronic device (see Figure 1), followed by sixty minutes of self-administered training, using the same device in the experimental group or conventional paper-and-pencil exercises in the control group. The cognitive intervention lasted two weeks, with five sessions per week, for a total of ten sessions.

The electronic device used in this study was a web-based platform called Neurotablet^®^, installed on a Samsung Galaxy tablet. Neurotablet^®^ is a sophisticated multi-platform system for cognitive rehabilitation, integrating robust hardware and software to provide a seamless therapeutic experience. Its software framework is powered by a custom-configured operating system optimized for neurorehabilitation applications. The system is designed with a strong emphasis on data security and regulatory compliance, adhering to both GDPR and HIPAA standards to ensure the confidentiality and integrity of patient information. Further details about the platform are provided in the Supplementary Materials.

Neurotablet^®^ includes customizable exercises divided into six cognitive domains: attention, memory, executive functions, language, perception, and neglect. Each exercise can be tailored by adjusting parameters such as stimulus type, quantity, and duration. The exercises are designed with varying levels of difficulty, which can be set by the therapist. The device also provides performance feedback through acoustic signals in case of errors and visual graphic summaries at the end of each exercise.

For this study, a neuropsychologist applied a standardized rehabilitation protocol, adjusting the difficulty level according to each patient’s abilities, with a specific focus on attention training. The neuropsychologist responsible for neuropsychological assessments was blinded to group allocation, while the neuropsychologist assigning the treatments was aware of the intervention. The exercises targeted subcomponents of attention (sustained, selective, and divided attention) and executive functions (planning, reasoning, and attentional shifting).

#### 2.4.1. Face-to-Face Treatment

Each morning, both groups received forty-five minutes of face-to-face cognitive rehabilitation using the electronic device. All patients performed the same types of exercises, as outlined in Table 1; however, specific exercise parameters (e.g., longer stimulus duration on screen) were individually customized by the therapist based on each patient’s cognitive profile. Each session consisted of forty minutes of active training and a five-minute break (see Table 1 for details on the exercises).

#### 2.4.2. Self-Treatment

##### Experimental Group

In the afternoon, the experimental group engaged in sixty minutes of self-guided cognitive training using the electronic device. Patients were instructed on how to operate the device and how to carry out the exercises appropriately, based on their individualized rehabilitation profiles and the tasks selected by the therapist. The interface displayed two blocks of tasks, with each exercise designed to last approximately fifteen minutes. A visual progress indicator on the screen helped patients monitor their advancement and recognize when an exercise was completed. Participants were encouraged to manage their time independently and to take short breaks between exercises as needed (see Figure 1). Each exercise was performed for fifteen minutes, with the option to take up to two breaks between tasks (see Table 2 for details on the exercises).

#### Control Group

In the afternoon, the control group engaged in sixty minutes of self-administered cognitive training using conventional paper-and-pencil materials. Each day, the therapist prepared a variety of exercises, including mazes, spot-the-difference tasks, crossword puzzles, target stimulus deletion, and dual-task activities, encouraging patients to engage with each type. Participants were invited to manage their time independently for each activity and to take breaks as needed, promoting self-regulation and autonomy throughout the training.

### 2.5. Outcome Measures

Two neuropsychologists, blinded to group allocation, evaluated the patients before (T0) and after the intervention (T1). To assess the participants’ cognitive profiles, a battery of neuropsychological tests targeting specific cognitive domains was administered.

#### 2.5.1. Primary Outcome

Selective visual attention was assessed using the Trail Making Test-Part A (TMT-A), which evaluates attentional capacity, particularly selective attention, as well as psychomotor speed and sequencing abilities. Shorter completion times indicate better performance [36].

#### 2.5.2. Secondary Outcomes

Executive functions were assessed through their core components: cognitive flexibility (shifting) using the Trail Making Test-Part B (TMT-B) [36], divided attention using the Dual Task [37], inhibitory control using the Stroop Test [38], and planning and problem-solving abilities using the Elithorn’s Perceptual Maze Test (EPMT) [39]. These tests and their results are discussed in detail in the following sections.

The TMT-B evaluates the ability to switch attention between two rules or tasks. The time required to complete the test is recorded, with shorter times indicating better performance [36].

The Dual Task assesses divided attention and consists of three two-minute trials: Span, Tracking, and Span and Tracking. In the first single task (Span), the patient repeats sequences of numbers of increasing length in the same order of presentation. In the second single task (Tracking), the patient is asked to cross out sequences of squares. In the third trial (Span and Tracking), both tasks are performed simultaneously. An index score (mu) is calculated by comparing performance on the single tasks with performance under the dual-task condition; higher scores indicate better performance [37].

The Stroop Color-Word Interference Test evaluates attention and executive functions, specifically the speed and accuracy of inhibiting automatic responses. The test includes three trials. In the first two trials (congruent condition), patients read characters printed in black and then identify colors from colored patches. In the third trial (incongruent condition), color-words are printed in a mismatched ink color, and patients must name the ink color rather than read the word. Two measures are recorded: interference time (Stroop-time) and interference errors (Stroop-errors), with lower scores indicating better performance [38].

The EPMT assesses attention and executive functions, specifically visuospatial planning, problem-solving, and strategic thinking. The test consists of eight trials, each presenting a mesh matrix with embedded black dots. Patients are instructed to trace a path through the matrix, touching a target number of black dots as quickly as possible. The number of correct responses is recorded (range: 0–16), with higher scores indicating better performance [39].

### 2.6. Statistical Analysis

In line with the exploratory nature of this proof-of-concept study, statistical analyses were conducted to provide preliminary insights that may support cautious speculation regarding the potential effects of the intervention, in addition to evaluating its feasibility and safety. Data were analyzed using IBM SPSS Statistics version 27.0 for Macintosh (IBM Corp., Armonk, NY, USA). The normality of data distribution was assessed using both the Kolmogorov–Smirnov and Shapiro–Wilk tests. Based on these results, appropriate statistical methods were applied according to the distribution of each outcome variable. Specifically, data that showed a normal distribution were analyzed using a two-way (2 × 2) mixed-design ANOVA, with “Time” (T0 vs. T1) as the within-subject factor and “Group” (experimental vs. control) as the between-subject factor. In contrast, data which did not meet the assumptions of normality were analyzed using non-parametric methods. For these variables, between-group comparisons at each time point (T0 and T1) were conducted using the Mann–Whitney U test, while within-group changes from T0 to T1 were assessed using the Wilcoxon signed-rank test. The significance level was set at *p* < 0.05 for all analyses.

## 3. Results

### 3.1. Baseline

**Figure 2:**
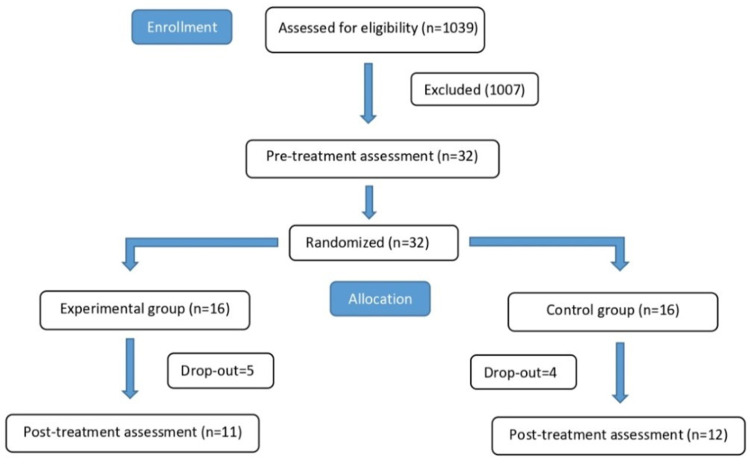
Flow diagram illustrating the structure and progression of the study.

From an initial sample of 1039 individuals referred to our center during the study period, 1007 were excluded due to compromised clinical conditions, hospitalization periods shorter than the time required to complete the trial, orthopedic diagnoses, departmental constraints related to the SARS-CoV-2 pandemic, or poor cooperation. The final study population consisted of 32 persons with stroke who met the inclusion criteria and were randomly assigned to either the experimental group (*n* = 16) or the control group (n = 16) (Figure 2). All participants were Italian. Nine patients were lost to post-treatment follow-up due to worsening general condition, early discharge or low motivation. Importantly, no dropouts were related to the protocol itself. The final sample included 11 patients in the experimental group and 12 in the control group. Baseline demographic and clinical characteristics are presented in Table 3, with no significant differences observed between groups. All patients who completed the intervention were able to carry out the sessions with minimal supervision during the two-week rehabilitation period.

### 3.2. Post-Intervention

Scores from the TMT-A, the Dual Task, and Stroop-time met the assumptions of normality and were analyzed using parametric methods. In contrast, scores from the TMT-B, Stroop-errors, and the EPMT did not meet normality assumptions and were therefore analyzed using non-parametric methods.

A post hoc power analysis was conducted to assess the actual statistical power achieved with the recruited sample. Based on an expected between-group difference of 19.02 s and a standard deviation of 60.60 s on the TMT-A, the achieved power with 11 and 12 participants per group was approximately 11.08%, using a two-sided t-test with α = 0.05.

#### 3.2.1. Primary Outcomes

Two-way ANOVA (2 × 2) of the TMT-A results revealed a principal significant effect of ‘Time’ (F1,21 4.708; *p* < 0.05) but not of ‘Group’ and of ‘Time × Group’ interaction. The mean difference between T0 and T1 was 30.22 s, with better performance at T1 (details in Table 4).

#### 3.2.2. Secondary Outcomes

ANOVA revealed a principal effect of ‘time’ for Dual Task (F1,21 12.135; *p* < 0.05) and Stroop-time (F1,20 4.55; *p* < 0.05) but not of ‘group’ and of ‘Time x Group’ interaction. The Dual Task score was higher after treatment (mean post-treatment score 90.46 (±18.18) vs. mean pre-treatment score 78.23 (±12.05). The Stroop-time score was lower after treatment (mean post-treatment score 40.53 (±25.27) vs. mean pre-treatment score 52.66 (±31.51). See Table 4 for further details.

For the outcome measures analyzed with non-parametric tests, the Mann-Whitney test on scores of TMT-B, Stroop-errors, and EPMT showed no statistically significant difference between the two groups. Within-group comparison showed changes between pre-and post-treatment scores on the three outcome measures for the experimental group. There was a difference in EPMT scores for the control group between T0 and T1 (details in Table 5).

## 4. Discussion

This proof-of-concept study aimed to evaluate the feasibility and safety of a semi-autonomous cognitive rehabilitation protocol using an electronic device (Neurotablet^®^) compared to conventional paper-and-pencil self-treatment in individuals with subacute stroke. While the original objective also included exploring potential cognitive benefits, the very low statistical power (11.08%) substantially limits the interpretability of the results and precludes definitive conclusions regarding efficacy. Consequently, the findings should be regarded as exploratory and interpreted with caution, focusing primarily on feasibility and safety. Despite this limitation, preliminary results suggest that the intervention was successfully integrated into standard rehabilitation schedules without disrupting other therapies. All participants who completed the protocol were able to carry out the sessions with minimal supervision, indicating that the approach is suitable for individuals in the early subacute phase post-stroke, a period in which patients are still recovering from acute neurological events but may already be capable of engaging in structured cognitive exercises. Importantly, no dropouts were attributable to the intervention itself, further supporting its safety, tolerability, and acceptability.

The Neurotablet^®^ platform offered several functional advantages over traditional approaches. The ability to deliver real-time feedback, such as acoustic signals for errors and visual summaries of performance, enabled patients to immediately recognize mistakes and adjust strategies, potentially fostering self-awareness and error monitoring. The system also allowed individualized adjustment of task parameters, meaning that exercises could be tailored in real time to each patient’s abilities and progress. Such adaptability may be particularly important in heterogeneous stroke populations, where cognitive impairments vary in severity, pattern, and recovery trajectory. Informal participant feedback further suggested that the Neurotablet^®^ was intuitive, motivating, and user-friendly, with patients appreciating the interactive features and structured interface. Although this feedback was anecdotal and not systematically assessed, it indicates promise for autonomous use in both inpatient and remote settings. The device’s usability is particularly relevant in telerehabilitation contexts, where remote programming and monitoring capabilities can extend cognitive care to individuals who may face barriers to in-person attendance, such as those with mobility limitations or those living in rural or underserved areas [16,19]. The semi-autonomous nature of the protocol, combining therapist-driven customization with patient-led execution, also has the potential to encourage metacognitive development, support adherence to therapy, and enhance independent time management skills.

Although between-group analyses did not reveal statistically significant differences, within-group improvements were observed in both conditions, particularly in visuospatial planning and problem-solving. In the experimental group, additional gains were noted in shifting attention and inhibitory control, domains that may benefit from the dynamic, time-sensitive nature of computerized cognitive training. These preliminary observations, while speculative given the small sample size, are consistent with findings from previous research reporting improvements in attention and executive functions following digital interventions [28,29,40]. The congruence between the present trends and earlier evidence suggests that the Neurotablet^®^ platform, and similar computerized systems, may have the capacity to target specific executive domains more effectively than static, paper-based tools.

From a methodological standpoint, several strengths reinforce the feasibility focus of this study. The intervention was implemented without extending the duration of treatment time, demonstrating that such a protocol can be accommodated within routine rehabilitation workflows. The use of a blinded evaluator minimized assessment bias, while standardized cognitive outcome measures ensured consistency across participants. The flexibility of the protocol to adapt to individual cognitive profiles also allowed for a patient-centered approach, aligning the difficulty and nature of the exercises with each individual’s strengths and weaknesses. The flexibility of the protocol to adapt to individual cognitive profiles also allowed for a patient-centered approach, aligning the difficulty and nature of the exercises with each individual’s strengths and weaknesses. This adaptability reflects core principles of person-centered rehabilitation, where interventions are tailored to the patient’s functional goals and personal context. In this framework, the Individual Rehabilitation Project (IRP) plays a central role, guiding the design of customized pathways that promote autonomy and active participation [41,42,43]. The semi-autonomous structure of the Neurotablet^®^ supports these aims by enabling self-management and dynamic adjustment of task parameters. Its capability to provide immediate, personalized feedback and to modulate exercise difficulty in real time represents a level of responsiveness that is difficult to achieve with conventional paper-and-pencil methods [33].

In addition, the interactive and engaging format of the computerized intervention appeared to increase patient motivation, an important factor in rehabilitation adherence. This finding, although anecdotal, resonates with previous literature suggesting that enjoyment and engagement can positively influence rehabilitation outcomes, even when objective performance measures show limited change. Such engagement may be especially critical in the subacute phase, when patients are still adapting to post-stroke limitations and may be at risk of reduced motivation due to fatigue, mood disturbances, or frustration.

The potential of computerized cognitive rehabilitation extends beyond the inpatient setting. In telerehabilitation applications, platforms like Neurotablet^®^ could enable therapists to remotely adjust exercise content and intensity, monitor progress in real time, and provide feedback without the need for physical presence. This could improve accessibility, continuity of care, and personalization, particularly for patients in geographically isolated areas or for those with transportation challenges [16,19].

However, the study’s limitations must be clearly acknowledged. The most critical constraint is the small sample size. A priori power analysis indicated that at least 80 participants (40 per group) would be required to achieve 80% power to detect a minimal detectable change of 19.02 s on the TMT-A [44]. With the current sample, the actual statistical power was only 11.08%, meaning the study was severely underpowered to detect meaningful between-group differences. Other limitations include the absence of follow-up assessments to evaluate the persistence of treatment effects over time, the lack of precise time monitoring during afternoon sessions in the control group, and the omission of formal usability or satisfaction assessments. The latter is particularly noteworthy, as informal feedback suggested high user satisfaction, but without validated measures, these perceptions cannot be quantified or compared. Furthermore, psychological and physical factors known to influence cognitive performance, such as anxiety, depression, pain, and fatigue, were not assessed [45,46]. These variables may have confounded the results, as mood and physical well-being can significantly affect engagement and cognitive performance in post-stroke populations. Additionally, evaluating baseline digital literacy could help optimize intervention delivery, ensuring that technological barriers do not impede participation or skew results. Understanding patients’ familiarity with technology would allow therapists to tailor training protocols and support materials, enhancing accessibility across diverse populations. In this context, it is also worth reflecting on the relatively low proportion of patients who were ultimately enrolled in the study. While only a small percentage of those screened met the inclusion criteria, this may be due to a combination of clinical, organizational, and contextual factors including the impact of the COVID-19 pandemic. It remains uncertain whether this figure accurately reflects the proportion of stroke survivors who could benefit from such interventions in routine practice. Broader implementation strategies and future studies in more flexible settings may help clarify the potential reach and generalizability of semi-autonomous cognitive rehabilitation protocols.

## 5. Conclusions

In conclusion, although the limited statistical power prevents this study from providing evidence of efficacy, it offers valuable insights into the feasibility and safety of a semi-autonomous computerized cognitive rehabilitation protocol for individuals in the early subacute phase post-stroke. The intervention was well tolerated, easily integrated into standard inpatient rehabilitation schedules, and perceived by participants as engaging and intuitive. These findings highlight the potential of digital tools in cognitive rehabilitation and reinforce the need for larger, adequately powered trials to assess their effectiveness. Future research should aim to recruit sufficient participants to ensure adequate power, incorporate follow-up assessments to explore the durability of treatment gains, and include validated measures of usability, satisfaction, and relevant psychological factors. Moreover, investigating the application of such protocols in telerehabilitation settings could enhance accessibility, continuity of care, and patient autonomy, ultimately broadening the reach of post-stroke cognitive rehabilitation.

## Figures and Tables

**Table 1:** In-person rehabilitation sessions conducted with the Neurotablet^®^ device.

Sessions	Exercises and Duration
SESSION 1 (day 1–2–3)	-15 min Selective Attention Letters Visual Disorders -10 min Selective Attention Aligned Numbers -15 min Flow Free
SESSION 2 (day 4–5)	-15 min Alternate Attention Letters -10 min Selective Attention Aligned Numbers -15 min Flow Free
SESSION 3 (day 6–7–8)	-15 min Single Uniform Attention -10 min Alternate Attention Visual Multiple Letters -15 min Split Screen Control/Inhibition
SESSION 4 (day 9–10)	-15 min Multiple Uniform Attention -10 min Control/Inhibition Acoustic Screen -15 min Stroop Arrows

Structure of face-to-face rehabilitation sessions using the computerized tool. The table illustrates the progression of cognitive exercises across sessions, targeting attention, visual processing, and executive functions through structured activities of varying durations.

**Table 2:** Afternoon rehabilitation exercises performed by the Experimental Group.

Sessions	Exercises and Duration
SESSION 1 (day 1–2–3)	-15 min Selective Attention Numbers Visual Disorders -15 min Flow Free -15 min Selective Attention Aligned Letters
SESSION 2 (day 4–5)	-15 min Alternate Attention Numbers -15 min Flow Free -15 min Selective Attention Aligned Letters
SESSION 3 (day 6–7–8)	-15 min Single Uniform Attention -15 min Split Screen Control/Inhibition -15 min Alternate Attention Visual Multiple Numbers
SESSION 4 (day 9–10)	-15 min Multiple Uniform Attention -15 min Control/Inhibition Acoustic Screen -15 min Stroop Arrows

Self-guided afternoon training sessions performed by the Experimental Group using the computerized tool. Each day, participants completed three 15-min cognitive exercises, selected by the therapist based on individual rehabilitation profiles. Patients were instructed to manage their own timing and were allowed to take short breaks between exercises.

**Table 3:** Baseline demographic and clinical data of the study sample.

Characteristic	EG *n* = 11	CG *n* = 12	*p*-Value (Z)
Sex—M/F	4/7	6/6	0.519 (−0.645)
Age—years	67 (±9.64)	66.92 (±10.52)	0.926 (−0.092)
Education level—years	10.36 (±5.04)	8.42 (±3.75)	0.297 (−1.043)
Stroke type—I/H	7/4	8/4	0.881 (−0.149)
Lesion-side (left/right/bilateral)	4/4/3	7/5/0	0.344 (−0.946)
Time from onset (days)	20.91 (±13.74)	23.92 (±19.59)	0.829 (−0.216)
Semantics subtest—OCS	3	3	1 (0)
Broken hearts subtest—OCS	34.82 (±10.71)	36.75 (±10.69)	0.44 (−0.772)
Spatial asymmetry—OCS	−0.64 (±1.63)	0.75 (±1.54)	0.061(−1.875)
Trails task subtest—OCS	8.63 (±4.47)	7.83 (±4.06)	0.64 (−0.468)

Demographic and clinical characteristics of the Experimental Group (EG) and the Control Group (CG). Data are presented as mean ± standard deviation).

**Table 4:** Outcome measures in the Experimental Group (EG) and the Control Group (CG).

Outcome Measure	Group	T0	T1
TMT-A (s)	EG	138.09 (±115.65)	102.27 (±52.55)
	CG	120.5 (±61.14)	95.42 (±56.39)
Dual Task (mu)	EG	72.18 (±13.91)	90.42 (±22.12)
	CG	84.49 (±13.16)	90.50 (±14.71)
Stroop-time (s)	EG	59.35 (±33.75)	43.6 (±26.56)
	CG	47.08 (±29.82)	37.98 (±25.02)

Mean and standard deviation of the outcome measures for the Experimental Group (EG) and the Control Group (CG) at baseline (T0) and post-intervention (T1).

**Table 5:** Wilcoxon signed-rank test for within-group changes between T0 and T1 in the Experimental Group (EG) and the Control Group (CG).

Outcome Measure	Group	T0	T1	*p*-Value (Z)
TMT-B (s)	EG	401.54 (±107.06)	342.73 (±158.28)	0.043 * (−2.028)
	CG	333.33 (±142.25)	309.92 (±214.37)	0.214 (−1.244)
Stroop- errors (0–30)	EG	7.9 (±8.03)	2.2 (±2.11)	0.038 * (−2.075)
	CG	8.58 (±7.83)	7.04 (±8.71)	0.168 (−1.378)
EPMT (0–16)	EG	9.45 (±3.25)	12.04 (±3.53)	0.049 * (−1.968)
	CG	10.68 (±3.88)	11.79 (±4.13)	0.046 * (−1.994)

Within-group comparisons between pre-treatment (T0) and post-treatment (T1) were conducted using the Wilcoxon signed-rank test in both the Experimental Group (EG) and the Control Group (CG). Data are expressed as mean ± standard deviation. * Statistically significant.

## Data Availability

The data associated with the paper are not publicly available but are available from the corresponding author on reasonable request.

## Supplementary Materials

The following supporting information can be downloaded at: https://www.mdpi.com/article/10.3390/neurosci6040109/s1, CE marking NT; Neurotablet_1 Manual.

## Abbreviations

The following abbreviations are used in this manuscript:

EG	Experimental Group
CG	Control Group
OCS	Oxford Cognitive Screen
TMT-A	Trail Making Test A
TMT-B	Trail Making Test B
EPMT	Elithorn’s Perceptual Maze Test
ANOVA	Analysis of Variance
SD	Standard Deviation
