# Supplementary material for: Self-Administered Cognitive Rehabilitation Using an Electronic Device in Subacute Stroke Patients: A Proof-of-Concept Study on Safety, Feasibility, and Preliminary Efficacy
Source: NeuroSci. 2025 Oct 30;6(4):109. doi: 10.3390/neurosci6040109 (PMC12642014; doi:10.3390/neurosci6040109)
Supplement: Supplementary file 1 [file neurosci-06-00109-s001.zip › neurosci-3848005-supplementary.pdf]

DISPOSITIVI MEDICI

Elenco dei dispositivi medici

Criteri di ricerca:
Denominazione fabbricante: **Neurab**
Codice fiscale fabbricante:
Partita IVA / VAT number fabbricante:
Codice nazione fabbricante:
Denominazione mandatario:
Codice fiscale mandatario:
Partita IVA / VAT number mandatario:
Codice nazione mandatario:
Tipologia dispositivo:
Identificativo di registrazione attribuito dal sistema BD/RDM:
Codice attribuito dal fabbricante:
Nome commerciale e modello:
Classificazione CND:
Descrizione CND:
Classe CE (valida solo per dispositivi medici di classe, impiantabili attivi e IVD):

Elenco dispositivi individuati

Dati aggiornati al:09/07/2017

DISPOSITIVO MEDICO/ASSEMBLATO									FABBRICANTE/ASSEMBLATORE				
TIPOLOGIA DISPOSITIVO	IDENTIFICATIVO DI REGISTRAZIONE BD/RDM	ISCRITTO AL REPERTORIO	CODICE ATTRIBUITO DAL FABBRICANTE/ASSEMBLATORE	NOME COMMERCIALE E MODELLO	CND	CLASSE CE	DATA PRIMA PUBBLICAZIONE	DATA FINE IMMISSIONE IN COMMERCIO	RUOLO AZIENDA	DENOMINAZIONE	CODICE FISCALE	PARTITA IVA/VAT NUMBER	NAZIONE
Dispositivo	1556214	N	001	NEUROTABLET	N010299 - DISPOSITIVI PER FISIOTERAPIA NEUROLOGICA - ALTRI	I - Classe I non sterile e senza funzioni di misura	07/06/2017		FABBRICANTE	NEURAB S.R.L.	02342090228	02342090228	IT

NEURAB
COGNITIVE RECOVERY

NEUROTABLET

IL TABLET PER LA RIABILITAZIONE NEUROPSICOLOGICA

MANUALE D'USO

La versione completa del manuale utente del Neurotablet è consultabile dal dispositivo stesso (dal menù a sinistra, sotto il nome del terapeuta) oppure dalla piattaforma areaneurab.com (dal menù in alto a destra, cliccando su "Loggato come xxx").

Neurab s.r.l.
Via Rialto, 73 – 38068 – Rovereto (TN) – Italy
Tel: +39 0464 436381 – Fax: +39 0464 400666
C.F. e P.IVA: 02342090228

Introduzione

1. Introduzione

1.1. Descrizione

Il Neurotablet è uno strumento elettronico per la somministrazione, a distanza e in presenza, di esercizi cognitivi per la riabilitazione neuropsicologica.

La confezione del Neurotablet contiene:

- **1** Samsung Tab A, con installato il software Neurotablet. Il Tablet è configurato per essere utilizzato esclusivamente entro le funzioni fornite dal Neurotablet.
- **1** custodia di gomma per tablet OtterBox
- **1** Cuffie Sennheiser 218

> Per utilizzare il Neurotablet è necessario avere un **account valido**. Per ottenere un account valido è necessario effettuare la registrazione con la **CHIAVE LICENZA**, che trovate stampata su un foglio all'interno della confezione.

1.2. Primo accesso e registrazione

> I seguenti passi per l'attivazione andranno compiuti **una sola volta**. Poi l'accesso sarà sempre effettuato esclusivamente con i vostri USERNAME e PASSWORD

Prima di effettuare il primo accesso al Neurotablet, assicurarsi di avere:

- Un Neurotablet
- Una connessione internet funzionante
- Una chiave licenza (si trova nella confezione)

> Se anche uno solo dei tre punti qui sopra non è disponibile, non sarà possibile procedere alla registrazione e quindi all'uso del Neurotablet!

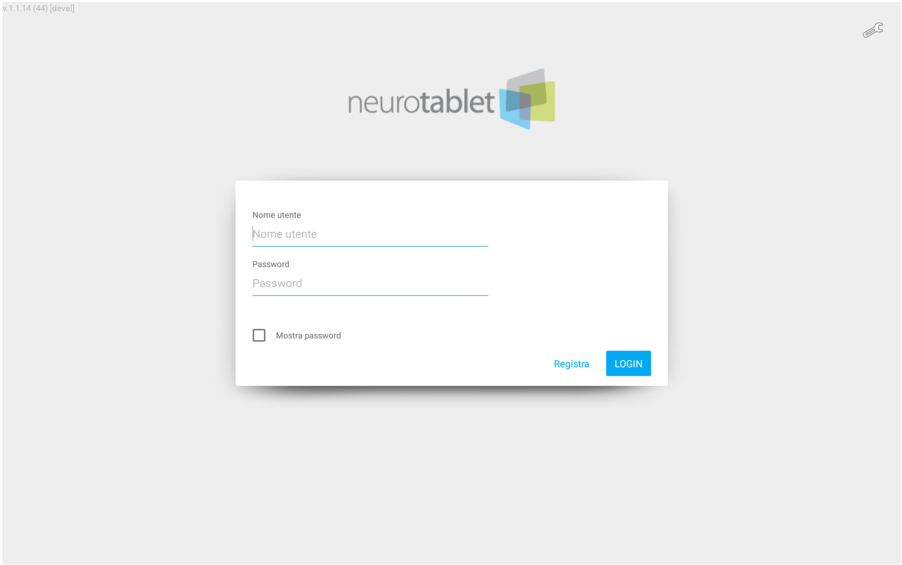

**Figure 1. La schermata di Login del Neurotablet.
In alto a destra si vede l'icona "settings".**

La schermata mostrata qui sopra è la prima che si visualizza quando si attiva il Neurotablet.

La registrazione consiste nella creazione di una coppia di **USERNAME-PASSWORD** che serviranno per l'uso del Neurotablet. Si può effettuare dalla pagina mostrata sopra tramite il tablet (connesso ad internet!) oppure tramite il sito ufficiale, **www.areaneurab.com**

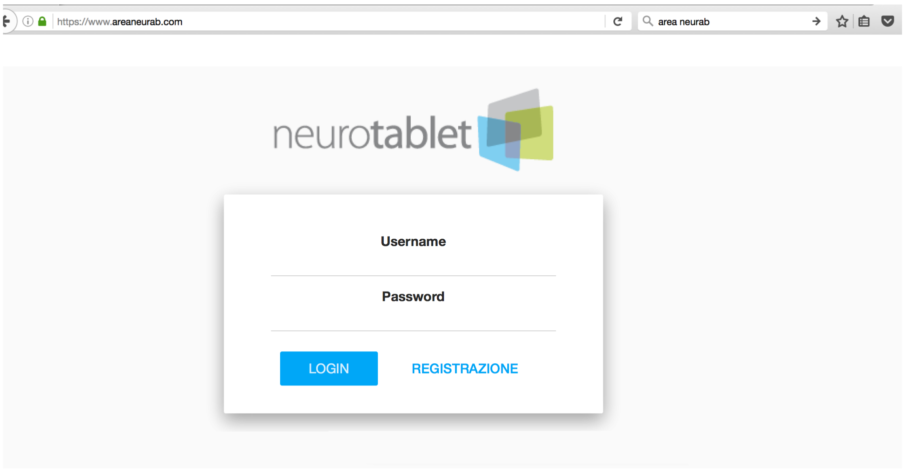

**Figure 2. La schermata di Login del sito ufficiale del Neurotablet.
Come si vede, l'interfaccia per l'accesso è molto simile.**

Far connettere il Neurotablet

> ⚠ Per utilizzare il Neurotablet è **NECESSARIO** che questo si colleghi ad internet **ALMENO UNA VOLTA** prima del primo login. Quando questa condizione non viene soddisfatta, **IL NEUROTABLET NON FUNZIONA**

Per connettere il Neurotablet ad una rete Wi-Fi è necessario seguire questi passi:

1) Andare sulla pagina iniziale del Neurotablet, e cliccare sulla **CHIAVE INGLESE IN ALTO A SINISTRA**

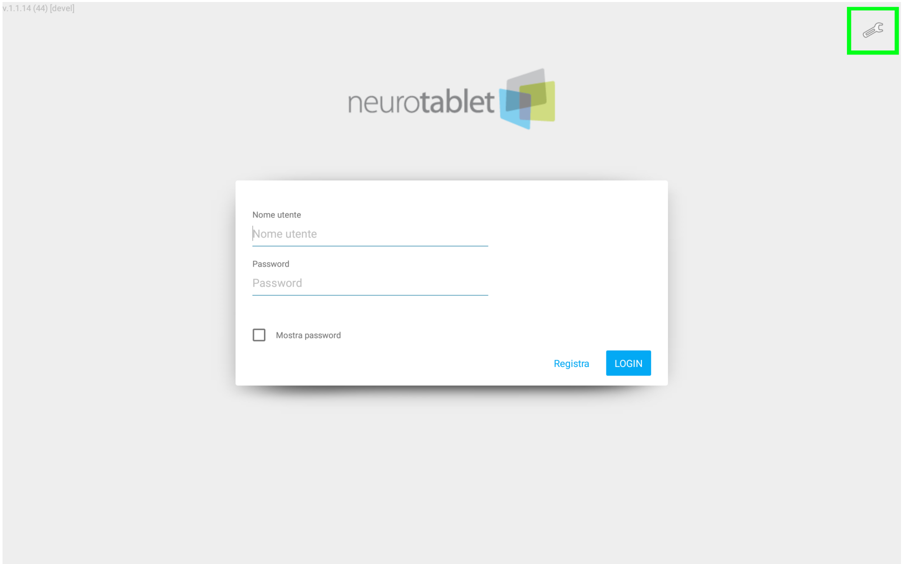

Figure 3. Per accedere alla schermata dei Settings di Android e dunque connettersi, premere sulla chiave inglese qui evidenziata.

2) A questo punto apparirà un menù simile a quello mostrato nella schermata qui sotto. Premere su "LA MIA ADSL" (che non necessariamente si chiamerà così, ma avrà il nome dell'ADSL di casa vostra) per connettersi.

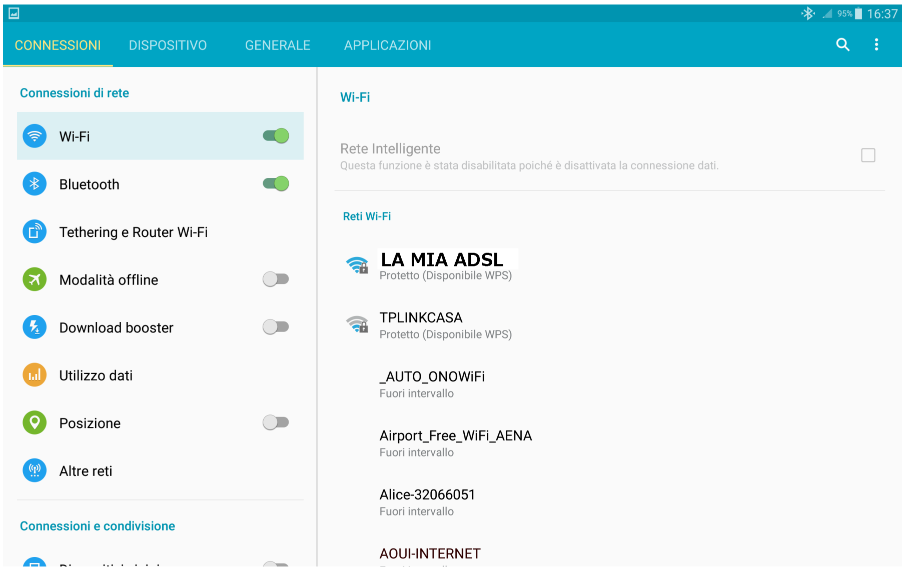

Figure 4. Un esempio del menù Wi-Fi che l'utente si troverà di fronte una volta cliccato sulla chiave inglese della pagina di login.

3) Se la vostra ADSL lo prevede, sarà necessario inserire la propria password. Se vi state connettendo ad un ADSL libera, questo passo non sarà necessario.

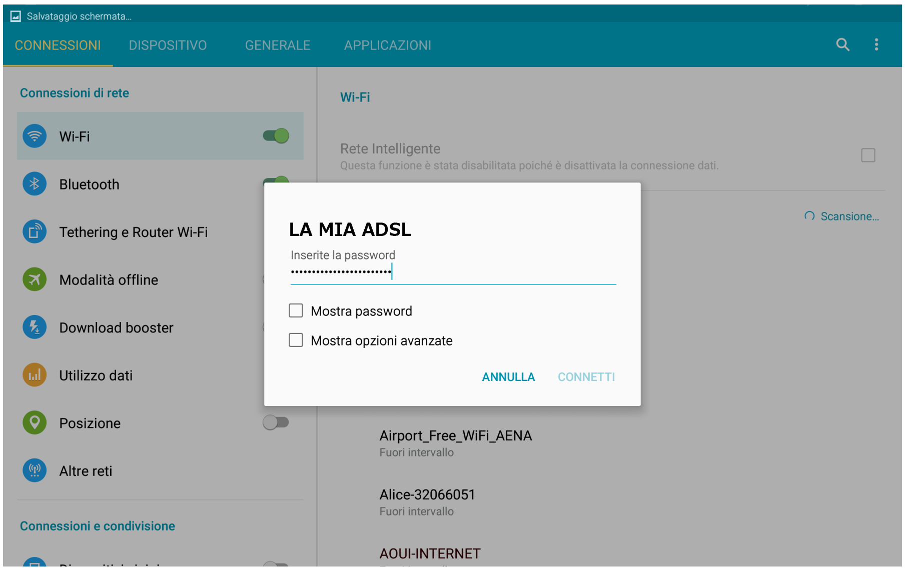

Figure 5. Inserite qui la password della vostra ADSL, se necessario, e premete "CONNETTI".

4) Se la connessione è andata a buon fine, vedrete il nome della VOSTRA ADSL colorato di verde, e la scritta connesso. Ora potete premere il tasto indietro e procedere con la registrazione del tablet.

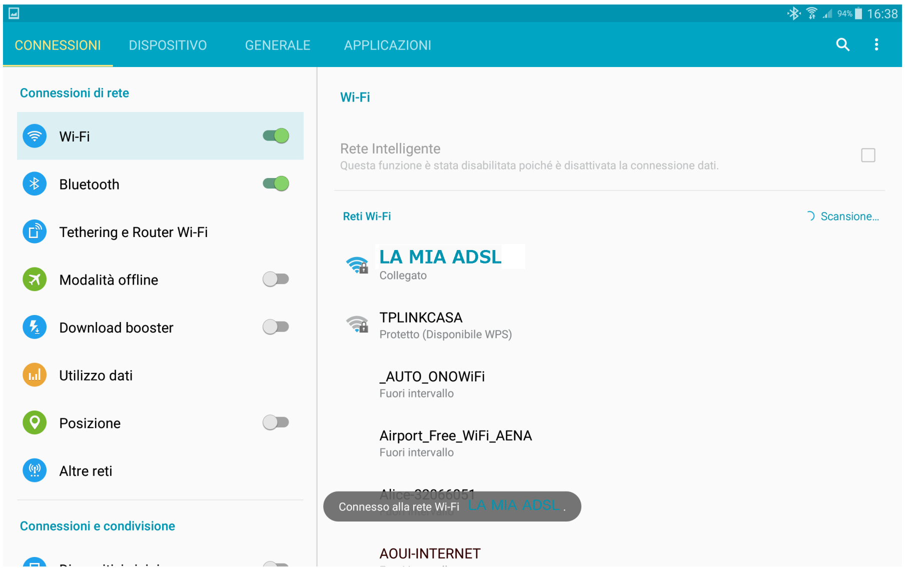

Figure 6. Questa è la schermata che indica l'avvenuta connessione del Neurotablet alla vostra Wi-Fi. Potete premere il tasto "indietro".

⚠ Una volta loggati, la connessione è possibile accedendo alla stessa schermata riportata qui sopra tramite il Menù a tendina

Inserire la propria CHIAVE LICENZA e creare il proprio account

Si può creare il proprio account sia dal web, tramite il sito http://www.areaneurab.com sia dal Neurotablet. Qui sotto vediamo come fare dal web oppure dal Neurotablet

⚠ La procedura di registrazione va svolta sul web o sul Neurotablet, sono equivalenti. **NON È NECESSARIO FARLO DA ENTRAMBE LE PARTI!** Inoltre, la seconda volta che la fate il codice non funzionerà più!

Dal web

1) Accedere al sito http://www.areaneurab.com e cliccare su **REGISTRAZIONE**

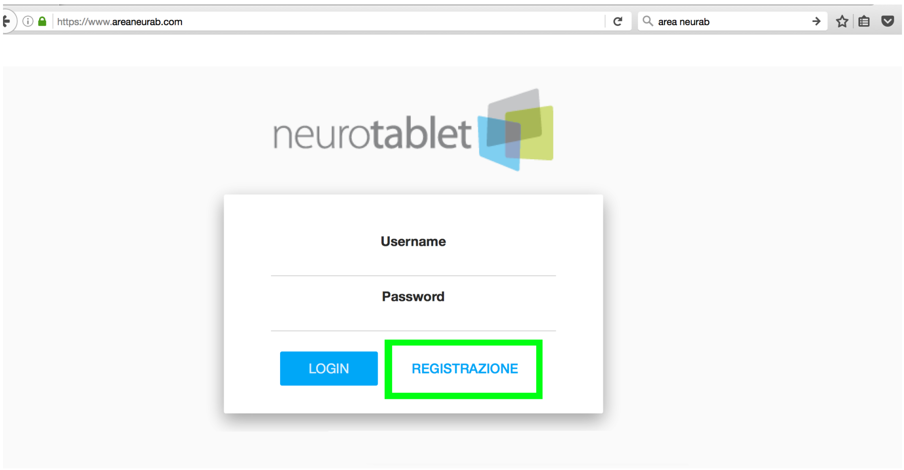

Figure 7. Questa è la schermata che si trova all'indirizzo http://www.areaneurab.com prima di effettuare il login. Per iniziare la procedura di registrazione, premere su "REGISTRAZIONE" come evidenziato in verde.

2) Una volta cliccato, la schermata apparirà così. Inserite nell'interfaccia qui sotto i vostri dati, dunque premete REGISTRAZIONE.

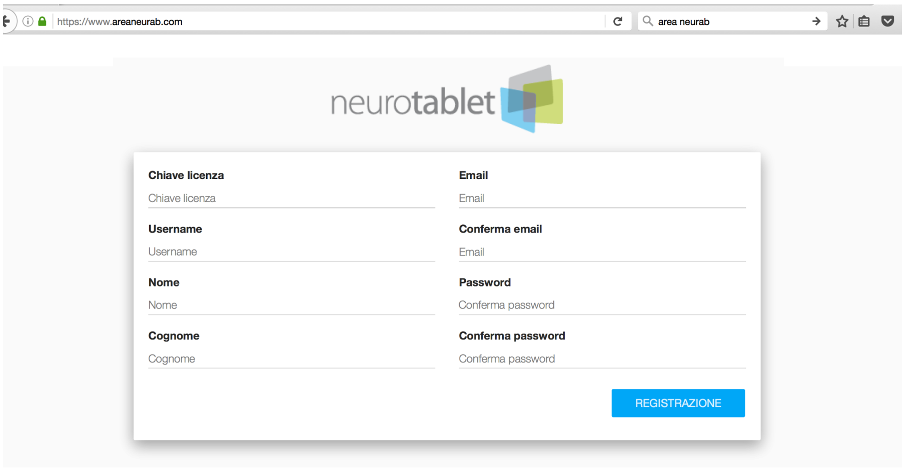

Figure 8. Questa è la schermata che si raggiunge dopo aver premuto su registrazione. Compilatela con i vostri dati, come spiegato sotto.

⚠ Il vostro **USERNAME** e la vostra **PASSWORD** sono **MOLTO IMPORTANTI**. Non dimenticateveli!

- **Chiave di Licenza**: la trovate nella confezione del Neurotablet. Digitate avendo cura di riportare minuscole e maiuscole, se ce ne sono.

- **Username**: sarà il vostro username: ovvero quello che, assieme alla password, inserirete ogni volta per effettuare l'accesso. Il programma infatti ha bisogno sia del vostro nome vero che di un altro nome, per associarvi ad una password. Potete scegliere il nome che volete, sia il vostro nome vero che un nome inventato.
- **Nome**: Il vostro nome di battesimo, o il nome della persona per cui è stato acquistato il Neurotablet
- **Cognome**: Il vostro cognome, o il cognome della persona per cui è stato acquistato il Neurotablet
- **Email**: il vostro indirizzo mail, assicuratevi che sia funzionante.
- **Conferma email**: digitate nuovamente il vostro indirizzo mail
- **Password**: scegliete una password che vi servirà, assieme al nome utente, per effettuare l'accesso le prossime volte.
- **Conferma Password**: Digitate nuovamente la password.

Prima di premere REGISTRA, verificate di aver scritto correttamente il vostro indirizzo mail. Questo è molto importante: se fornite un indirizzo mail non funzionante non sarà poi più possibile recuperare la vostra password nel caso la dimenticaste!

Dal Neurotablet

Per registrarsi tramite il neurotablet è **NECESSARIO** che questo sia connesso ad internet.

1) Per registrarsi tramite il Neurotablet, premere sul tasto "REGISTRA" come evidenziato.

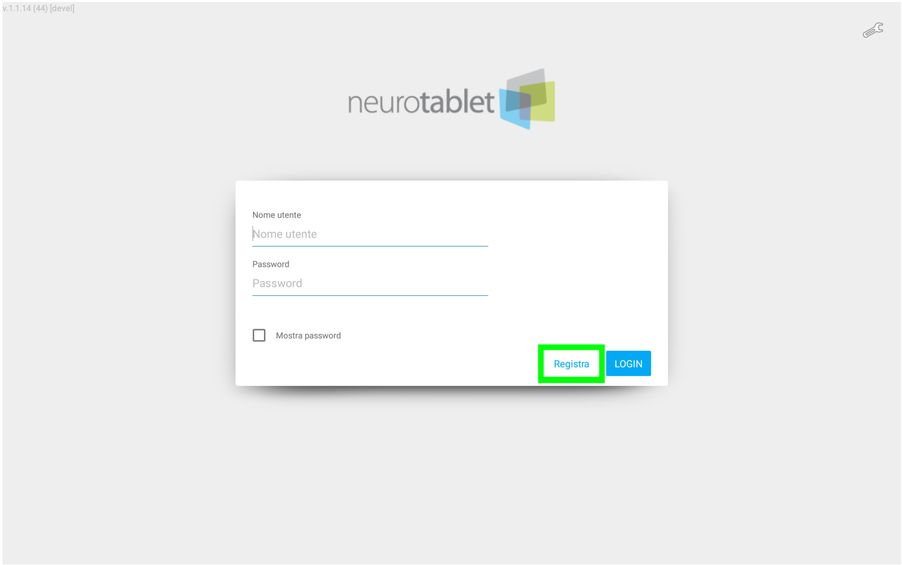

Figure 9. Questa è la schermata di login del Neurotablet, con il tasto REGISTRA evidenziato.

2) Inserire qui sotto i seguenti campi:

> ⚠ Il vostro **USERNAME** e la vostra **PASSWORD** sono **MOLTO IMPORTANTI**. Non dimenticateveli!

- **Chiave di Licenza**: la trovate nella confezione del Neurotablet. Digitate avendo cura di riportare minuscole e maiuscole, se ce ne sono.
- **Username**: sarà il vostro username: ovvero quello che, assieme alla password, inserirete ogni volta per effettuare l'accesso. Il programma infatti ha bisogno sia del vostro nome vero che di un altro nome, per associarvi ad una password. Potete scegliere il nome che volete, sia il vostro nome vero che un nome inventato.
- **Nome**: Il vostro nome di battesimo, o il nome della persona per cui è stato acquistato il Neurotablet
- **Cognome**: Il vostro cognome, o il cognome della persona per cui è stato acquistato il Neurotablet
- **Email**: il vostro indirizzo mail, assicuratevi che sia funzionante.
- **Conferma email**: digitate nuovamente il vostro indirizzo mail
- **Password**: scegliete una password che vi servirà, assieme al nome utente, per effettuare l'accesso le prossime volte.
- **Conferma Password**: Digitate nuovamente la password.

Prima di premere REGISTRA, verificate di aver scritto correttamente il vostro indirizzo mail. Questo è molto importante: se fornite un indirizzo mail non funzionante non sarà poi più possibile recuperare la vostra password nel caso la dimenticaste!

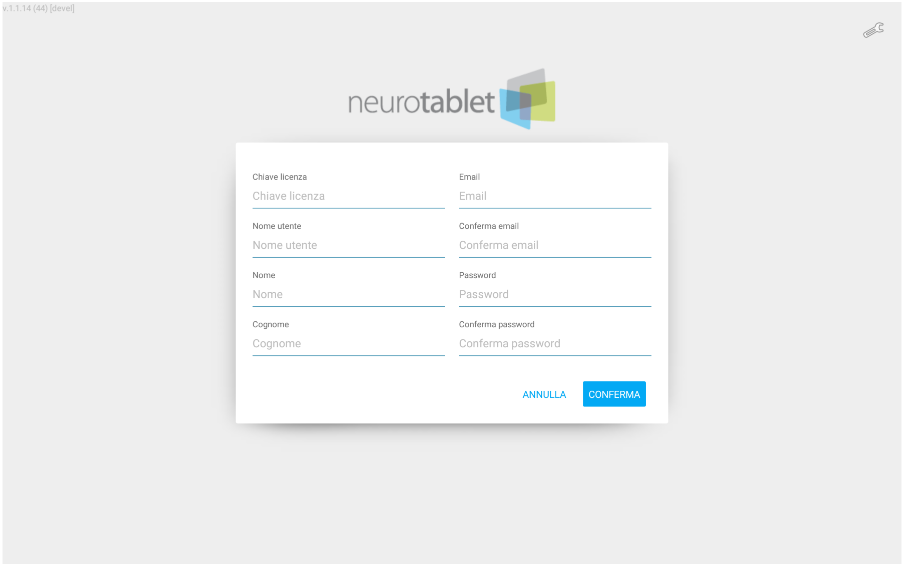

Figure 10. Questa è la finestra in cui inserire i dati di registrazione.

Registrazione confermata:

La registrazione andata a buon fine sarà confermata da un messaggio del Neurotablet, come quello riportato qui sotto. D’ora in poi potrete effettuare il login tramite la procedura di login standard.

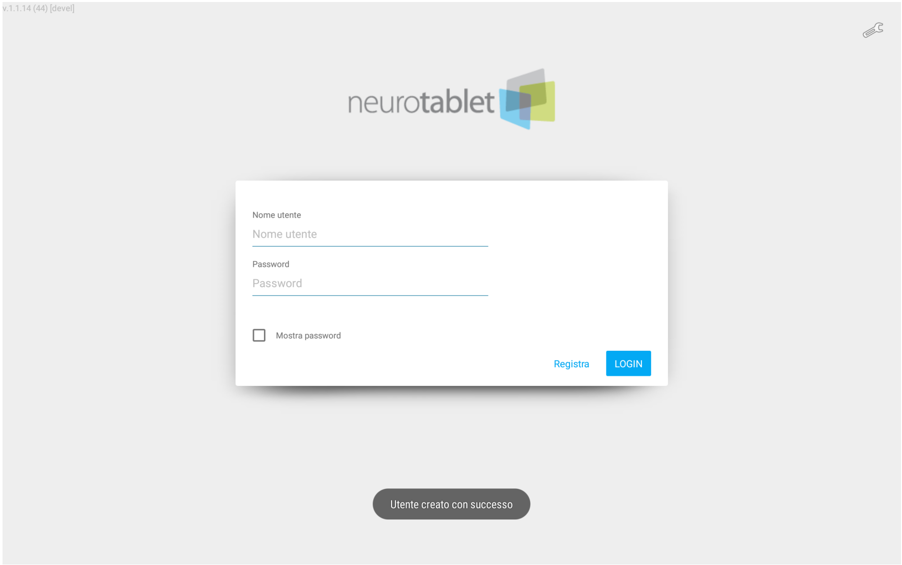

Figure 11. Un esempio di schermata mostrata per una registrazione andata a buon fine.

1.3. Schermata Principale

Appena effettuato l'accesso, vi troverete di fronte alla seguente schermata principale.

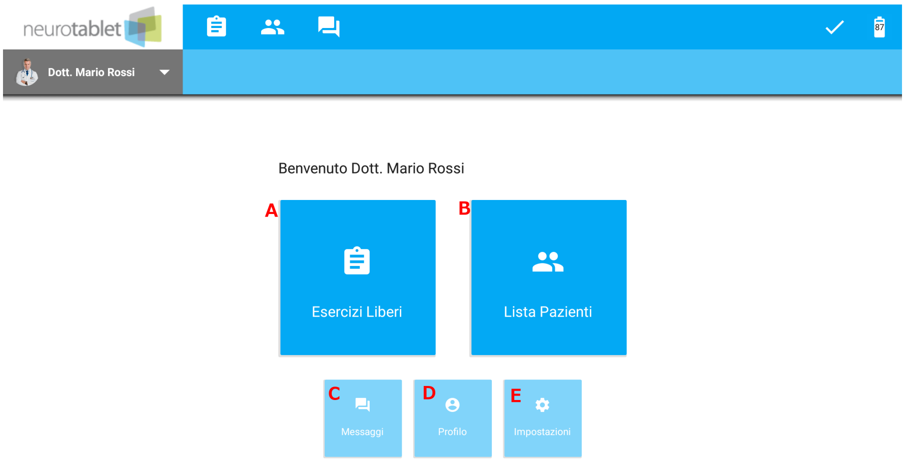

Figure 12. La schermata principale del Neurotablet

La schermata contiene i seguenti elementi:

- A: **Esercizi liberi.** Questo pulsante permette di accedere alla lista di tutti gli esercizi, e di esercitarsi come terapeuta

- B: **Lista pazienti.** Questo pulsante permette di accedere alla lista pazienti, e da lì selezionare un paziente da far allenare liberamente o sul proprio percorso terapeutico.
- C: **Messaggi.** Da qui potete scambiare messaggi di testo con tutti i vostri pazienti che posseggono un Neurotablet con abbonamento valido
- D: **Profilo** Dove modificare i propri dati personali e verificare la validità del proprio account e la durata dell'abbonamento
- E: **Impostazioni** Dove modificare il proprio profilo e altre impostazioni generiche del Neurotablet.

> La differenza tra gli esercizi visualizzati a partire da "esercizi liberi" e invece da "lista pazienti" → "paziente" è che i DATI prodotti dagli esercizi saranno assegnati al paziente specifico che si è selezionato.
> Se invece si sceglie "esercizi liberi" non ci sarà modo di risalire ai dati del paziente specifico, perché il Neurotablet non saprà a quale paziente associare i dati!!

1.4. Barra menù

Inoltre, sopra vi è una barra che ha la seguente composizione:

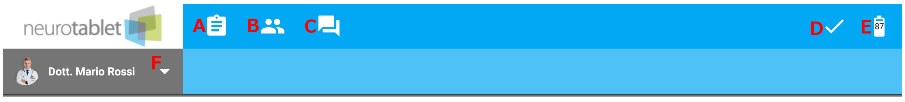

Figure 13. La barra menù superiore del Neurotablet

- A: **Esercizi liberi.** Questa icona permette di accedere alla lista di tutti gli esercizi, e di esercitarsi come terapeuta
- B: **Lista pazienti.** Questa icona permette di accedere alla lista pazienti, e da lì selezionare un paziente da far allenare liberamente o sul proprio percorso terapeutico.
- C: **Messaggi.** Apre l'interfaccia per scambiare messaggi di testo con tutti i vostri pazienti che posseggono un Neurotablet con abbonamento valido
- D: **Indicatore di sincronizzazione** L'indicatore di sincronizzazione mostra se i dati presenti sul tablet sono sincronizzati a quelli sul server
- E: **Indicatore carica residua** Mostra la carica residua della batteria
- F: **Menù a tendina** Premendo sul triangolino appare un menù con diverse funzioni, tra cui quella delle impostazioni dispositivo.

1.5. Indicatore di sincronizzazione

Il Neurotablet invia i dati in forma criptata ed a norma di legge di tutti i pazienti ai server Neurab ogni volta che può. Questo serve per avere una corrispondenza tra i dati visti sulla piattaforma web e sul tablet stesso.

Indicatore OK

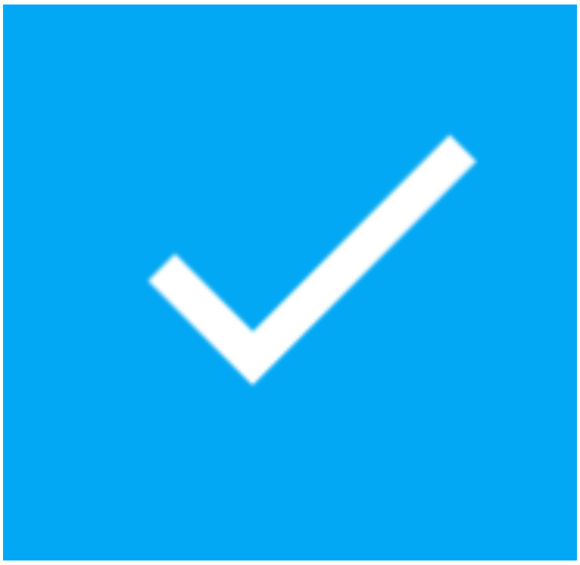

Figure 14. L'indicatore di sincronizzazione su "ok". Se lo vedete così, va tutto bene.

L'indicatore di sincronizzazione è sempre presente in alto a sinistra, ed indica appunto se i dati presenti sul Neurotablet corrispondono a quelli presenti sui server di Neurab.

La sincronia è garanzia di buon funzionamento della piattaforma, e del Neurotablet qualora si vogliano visualizzare dati e sessioni distanti nel passato.

Premendo sull'indicatore in modalità "ok", si apre il pop-uè rappresentato qui sotto.

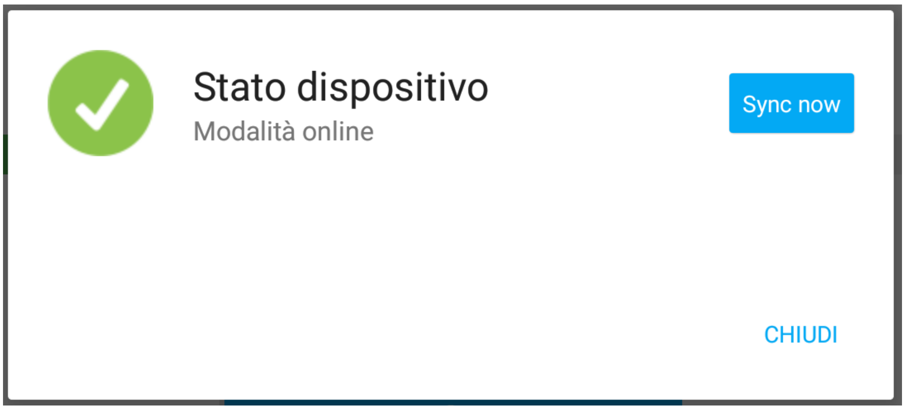

Figure 15. L’indicatore segna tutto OK.
Premendo esce un pop-up che lo conferma.

Problemi di sincronizzazione

Se invece vi sono problemi di sincronizzazione, l’indicatore appare così.

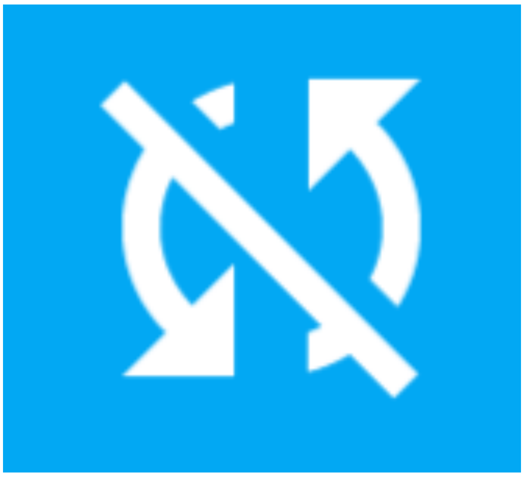

Figure 16. L’indicatore segnala problemi di sincronizzazione.

In tal caso, premendoci sopra, potrebbe apparire un pop-up che segnala l’errore, ed elenca le cose che NON è riuscito a sincronizzare.

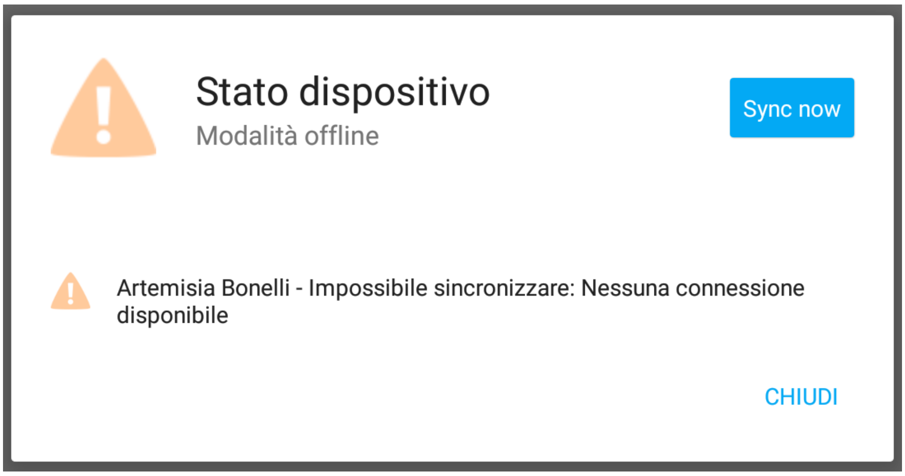

Figure 17. Un esempio di finestra che mostra problemi di sincronizzazione.

Quando siete in questa situazione, è sempre utile provare a fare "Sync Now". Se era soltanto un problema temporaneo, l’icona dovrebbe diventare come sotto, per poi tornare a mostrare "tutto ok"

Sincronizzazione in corso

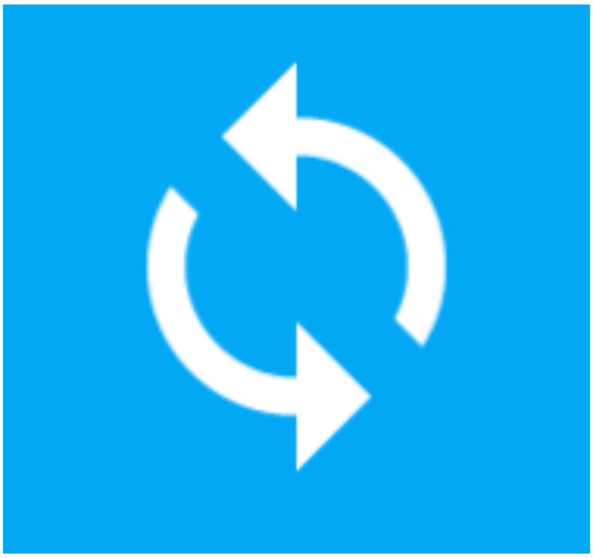

Figure 18. Un esempio di icona che mostra la sincronizzazione in corso.

Premendoci sopra, vedrete apparire un pop-up che mostra di volta in volta che cosa sta venendo sincronizzato.

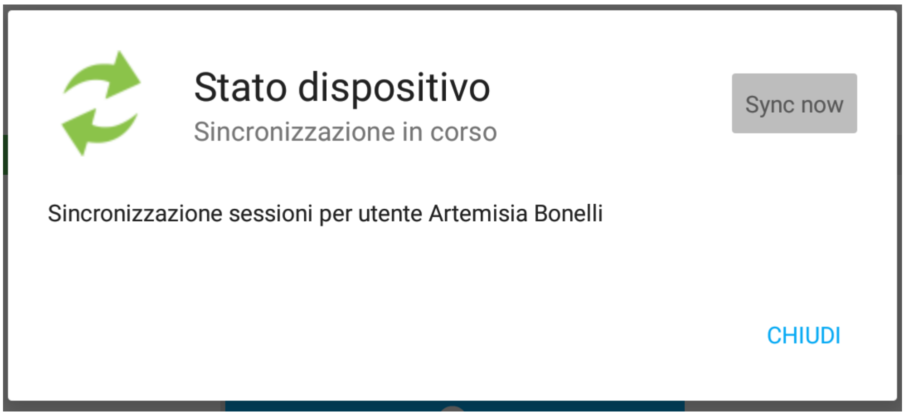

Figure 19. Un esempio di pop-up che mostra la sincronizzazione in corso.

Se anche questo non risolve il problema, suggeriamo di rivedere la procedura "Connettere ad Internet il Neurotablet" oppure contattare l’assistenza.

Confitto nei file di log

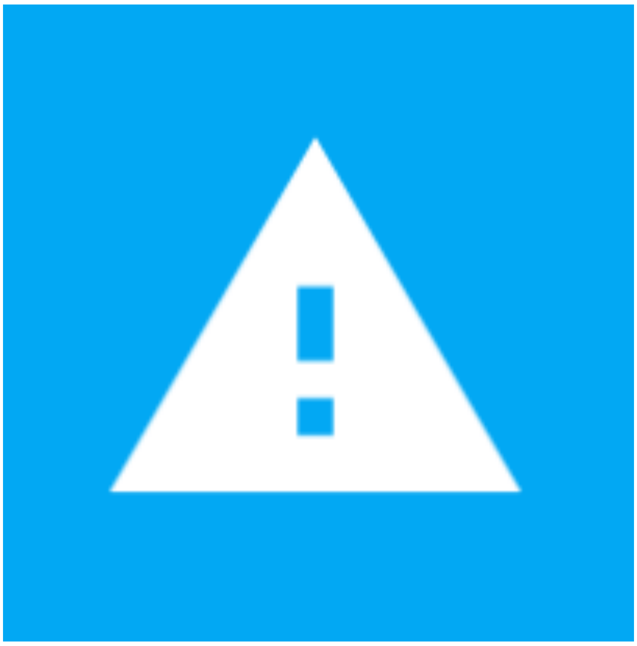

Figure 20. Quando vi sia un conflitto nei file di log, l’indicatore sulla barra appare così.

Vi sono casi particolari in cui si può verificare un conflitto tra i dati che ci sono sul tablet ed i dati che ci sono sui server. Questo solitamente accade perché si è giocato con **DUE** tablet differenti però con lo stesso **ACCOUNT**, cioè effettuando l’accesso con gli stessi login e password nello stesso periodo temporale e su due tablet diversi.

ES: Il terapeuta ha un suo Neurotablet, con una lista di tutti i pazienti tra cui la paziente Artemisia Bonelli. Questa ha pure il suo tablet, e ci gioca a casa sua: però si dimentica di connetterlo alla sua Wi-Fi. Artemisia un giorno si reca dal terapeuta, e gioca alcune sessioni con il tablet del terapeuta, ma con il proprio **profilo paziente**. Poi torna a casa, e si ricorda di connettere il Neurotablet.

Appena lo connette, il Neurotablet prova a inviare i propri dati ai server; ma trova quelli che Artemisia ha prodotto nello studio del terapeuta. Ora deve decidere quali dati tenere: quelli di casa di Artemisia, oppure quelli dello studio del terapeuta? Entrambi vengono dallo stesso account (Il paziente **Artemisia Bonelli**).

Per risolvere il conflitto, è necessario premere sull'indicatore di conflitto. Facendolo, apparirà un pop-up simile a quello qui sotto. Ci informa che vi è un conflitto di sincronizzazione.

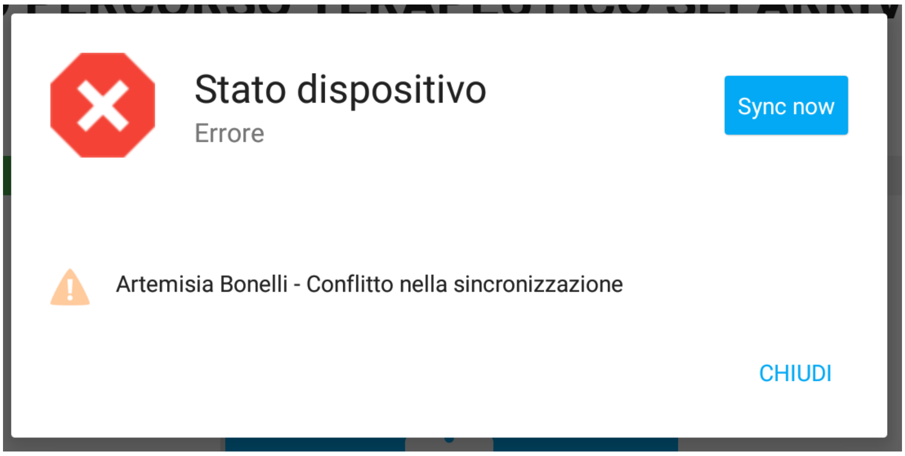

Figure 21. Premendo l'indicatore di conflitto, appare un pop-up simile.

Questo pop-up mostra tutti Premendo sul conflitto di sincronizzazione (in questo caso, premendo su "Artemisia Bonelli - Conflitto nella sincronizzazione) si attiva la procedura di risoluzione conflitti.

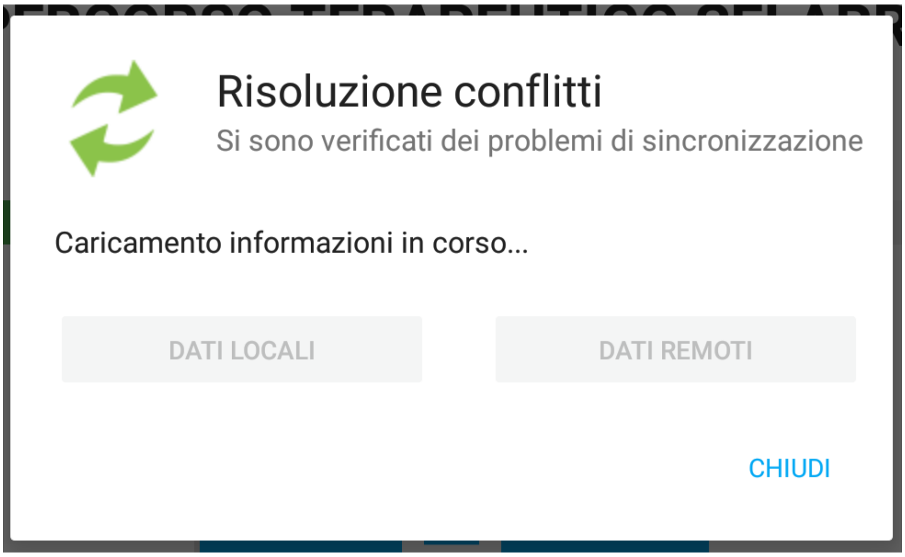

Figure 22. Premendo su "gestisci conflitti" si visualizza questa finestera.

Il programma fornisce quindi un pop-up che mostra gli elementi di conflitto. Vengono indicati gli esercizi e le sessioni che hanno prodotto conflitto, ovvero che non corrispondono tra Neurotablet e piattaforma web.

L’utente procede quindi a risolvere i conflitti, decidendo quali dati tenere: i dati del tablet, oppure i dati remoti, ovvero i dati presenti sui server di Neurab.

> Scegliendo una delle due alternative (locale vs. remoto) si **CANCELLANO IN MANIERA DEFINITIVA** i dati presenti nell’altra. Si tratta di una **DECISIONE IRREVERSIBILE**, quindi valutate bene **QUALI** dati tenere e quali cancellare!

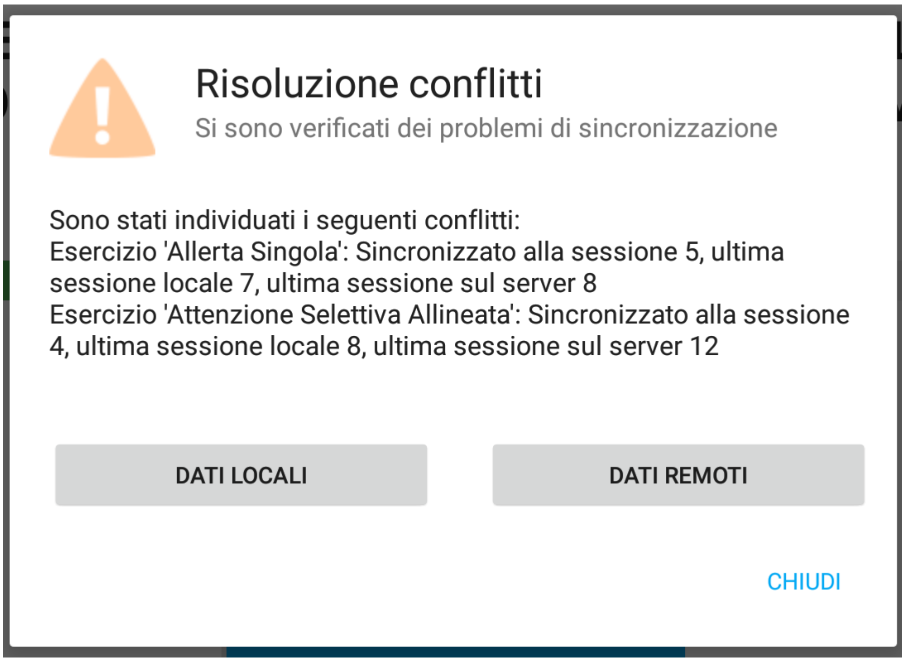

Figure 23. Premendo su "gestisci conflitti" si visualizza questa finestra.

1.6. Il menù a tendina

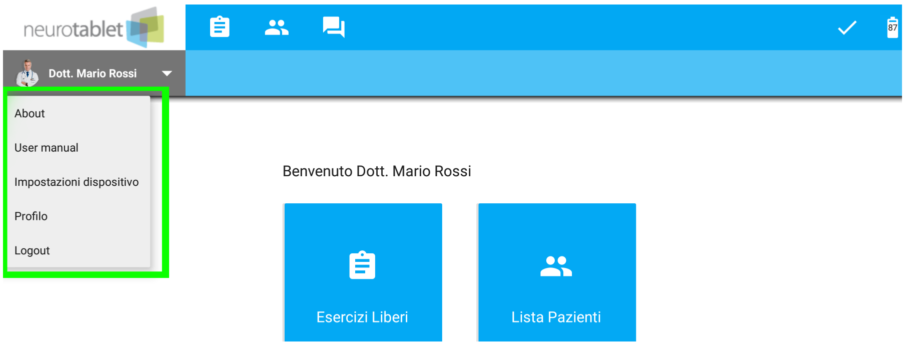

Figure 24. Premendo sul triangolino accanto al nome del terapeuta, si visualizza il menù a tendina.

Premendo il triangolino accanto al nome utente appare il menù a tendina. Questo presenta quattro voci:

- **About:** Informazioni sulla versione del software e sugli autori.
- **User manual:** accesso a questo manuale
- **Impostazioni dispositivo:** si accede ad un menù Android di default sul dispositivo. Qui è possibile verificare la connettività del tablet e tutte le altre opzioni hardware.
- **Logout** Il terapeuta esce dal suo account. Diventa possibile loggarsi con altri account.

Attenzione: dopo aver effettuato il logout sarà **impossibile effettuare il login se non si è online!**

2. Gli esercizi

2.1. Le Aree

Gli esercizi del Neurotablet sono divisi in 6 aree. Le aree corrispondono alla funzione cognitiva e in un caso (Neglect) alla patologia specifica da riabilitare.

- Attenzione
- Neglect
- Memoria
- Percezione
- Funzioni Esecutive
- Linguaggio

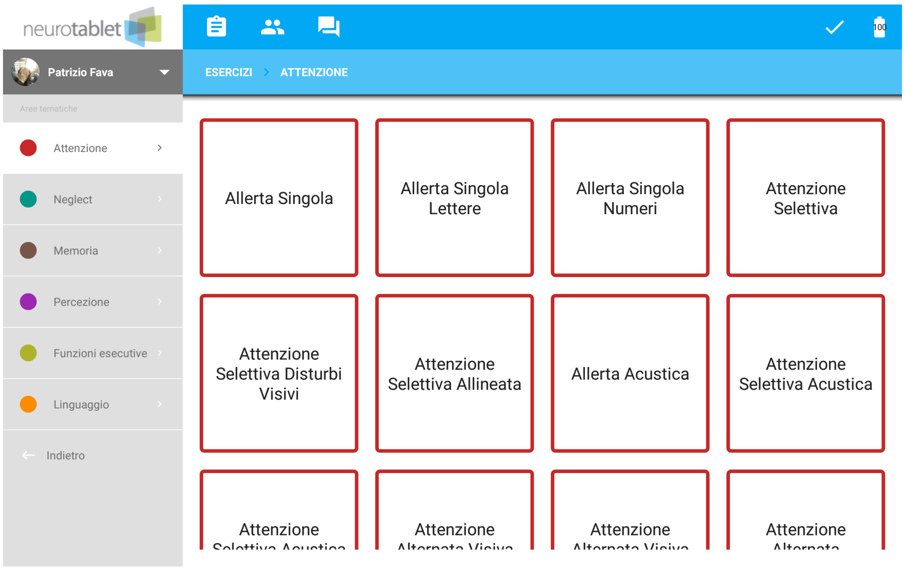

Figure 25. La schermata esercizi.

Le aree sono lungo la barra grigia a sinistra. Selezionandone una, appaiono a destra gli esercizi presenti in quell'area

2.2. Struttura gioco

Ciascun esercizio è strutturato nell'esecuzione in:

- Trial
- Sessioni

Un trial è una prova singola, che può essere giusta o sbagliata, contenuta all'interno di una sessione. In tutti gli esercizi, si può capire a quale trial si sia arrivati osservando la barra di progressione in alto.

Figure 26. La barra progressione è sempre in alto, ed è presente SOLO durante l'esecuzione del gioco.

I rettangolini in cui è divisa la barra sono inizialmente tutti vuoti: col procedere dei trial, si riempiono uno alla volta.

> Osservando la barra in alto si può capire, durante il gioco, a che punto si è della sessione e quanto manca alla fine.

Una sessione è una sequenza di trial, per la quale si seleziona un livello di difficolta ed altri settings, che rimangono tali fino alla fine della sessione.

Quando si entra nei menù, e si inizia un gioco, si intende che si inizia una sessione di gioco.

Nuovo/Continua

Subito dopo aver selezionato un esercizio, si viene posti di fronte alla schermata Nuovo/ Continua.

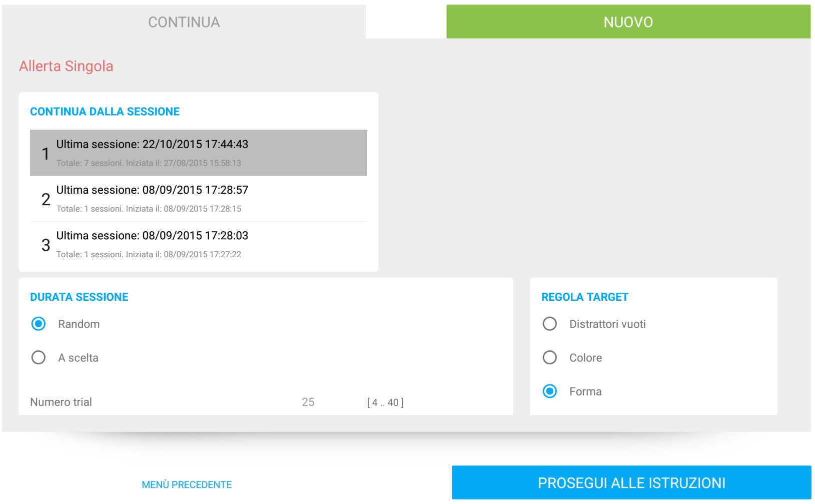

Figure 27. La schermata Nuovo/Continua.

Questa schermata permette di decidere se cominciare una nuova sessione, stabilendo livello e parametri da capo, oppure continuare una sequenza di sessioni precedenti.

La sezione dedicata ai settings è, come si vede, divisa in due. Nei Settings "Continua" si trovano tutti i parametri che possono essere modificati nella schermata "Continua", quindi continuando da una sequenza di sessioni iniziata precedentemente. Nei Settings "Nuovo" si trovano tutti i parametri che possono essere modificati prima di iniziare una nuova sessione.

I settings "Continua" sono un sottoinsieme dei settings "Nuovo".

> Prova a far scorrere il pallino nero sulla barra dei livelli: vedrai cambiare i parametri in tempo reale. Guardandoli, puoi capire quali stimoli e che tempo di presentazione troverai nella sessione che stai per iniziare.

Box Continua dalla sessione

A destra è possibile selezionare da dove si vuole proseguire. A sinistra invece, si decide per una nuova sessione. Se si sceglie di riprendere da una vecchia sessione, bisogna selezionare quale.

Le sessioni sono archiviate in forma di **SEQUENZE** di sessioni. Una sequenza di sessioni è una serie di sessioni che sono state raggiunte premendo "continua" alla fine della sessione precedente.

Perché scegliere una sequenza di sessioni

La maggior parte dei giochi del Neurotablet contiene delle funzioni che si adattano alle performance del paziente, assestandosi automaticamente su una difficoltà ottimale.

In media, le funzioni di adattamento ci mettono circa tre-quattro sessioni per "sintonizzarsi" sulle performance specifiche del paziente. In questo contesto, può essere utile iniziare con un paziente un esercizio ad un dato livello, e poi, se il livello era adeguato ed il setting ed il compito altrettanto - continuare sempre con quella serie di sessioni, in modo da avere una crescita "ottimale" della difficoltà del compito.

Naturalmente questa è solo UNA modalità di gioco possibile: il terapeuta può anche decidere di settare tutti i parametri di ciascun esercizio a mano ogni volta, o solo alcuni, e quindi personalizzare ancora di più la terapia.

Contenuto del box

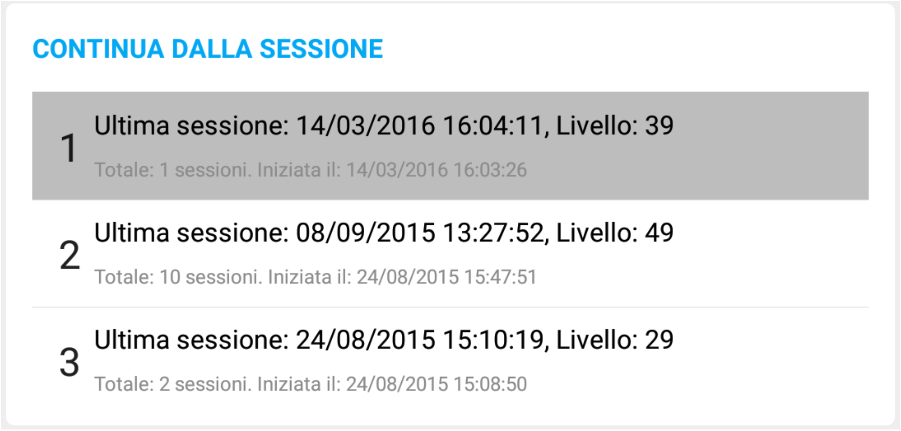

Figure 28. Un dettaglio della visualizzazione delle sequenze delle sessioni precedenti.

Il Box contiene un elenco di sequenze di sessioni, concatenate dalla pressione del tasto continua.

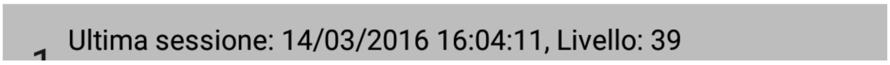

Figure 29. La riga ultima sessione in nero.

- La riga "ultima sessione" in nero riporta:
  - # La data di fine dell’ultima sessione. *Nell’esempio: (14/03/2016)*
  - # L’ora di fine dell’ultima sessione *Nell’esempio: (16:04)*
  - # Il livello a cui è arrivato il paziente giocando *Nell’esempio: Livello 39*

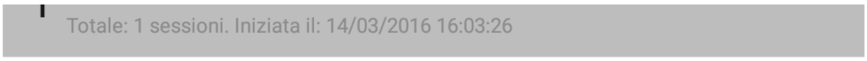

Figure 30. La riga ultima sessione in grigio.

- La riga in grigetto, in piccolo, sotto, riporta invece:
  - # La lunghezza totale in sessioni della sequenza
  - # La data di inizio della prima sessione
  - # L’ora di inizio della prima sessione.

Il livello da cui partirà il paziente è quello mostrato sulla riga nera. In questo caso il 39

Play/Pausa

Si può fare "pausa" in ogni momento premendo il tasto indietro accanto al tasto "home". Il tasto indietro è a fianco dello schermo, ed è indicato da una freccia ad "U".

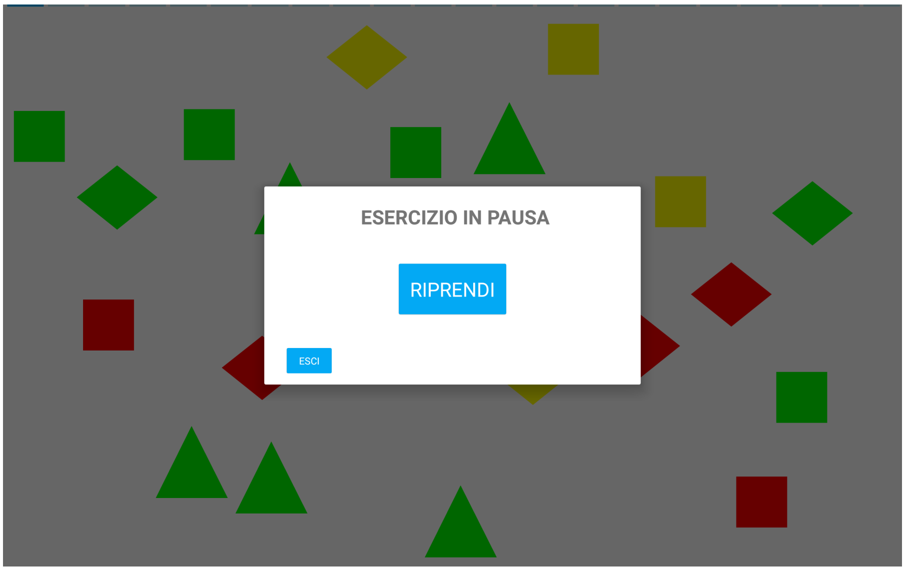

Figure 31. Il Pop-up che appare quando si preme "indietro".

Facendo pausa si allunga di un trial la sessione. Il trial che era presentato durante la pressione del tasto "indietro", infatti, viene eliminato dal conteggio, e ne viene aggiunto uno in più alla fine.

Grafici di fine sessione

Alla fine di ogni sessione saranno visualizzati due grafici:

Il grafico torta dell'accuratezza

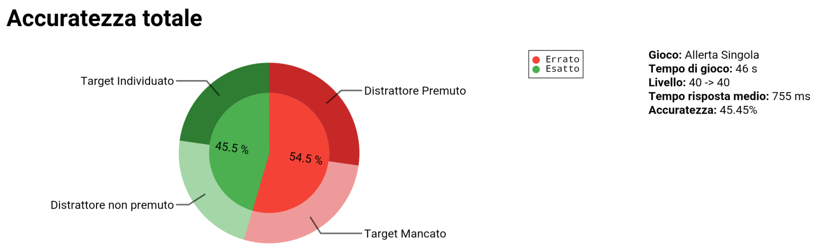

Figure 32. Un esempio di grafico torta.

Il grafico torta mostra una sintesi dell'accuratezza e degli errori commessi dal paziente in quella sessione.

Ciascun esercizio ha le sue specifiche categorie, che vengono riportate nella legenda contestuale. In generale, però:

- Lo strato esterno della torta indica l'accuratezza (rosse=errate/verdi=esatte) generale della sessione
- Lo strato interno della torta indica le percentuali di categorie di risposte esatte e di risposte errate.

Il grafico a barre dei tempi di reazione

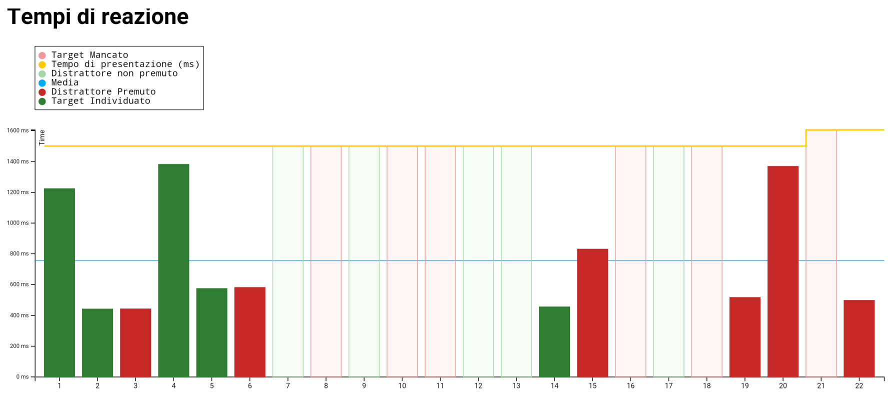

Figure 33. Un esempio di grafico a barre.

Il grafico a barre mostra i tempi di reazione e l'accuratezza specifica per ciascun trial. Ciascun esercizio ha le sue categorie contestuali, ma in generale:

- Ogni barra corrisponde ad un trial

- L'altezza della barra corrisponde al tempo di presentazione del trial
- Il colore della barra la categoria di risposta (esatta/errata)
- Premendo sulla barra appare un pop-up (non in tutti gli esercizi) che mostra ulteriori dettagli. Il tipo e la quantità di dettagli dipendono dall'esercizio stesso.

> Se tocchi le barre del grafico, vedrai apparire un pop-up con dettagli ulteriori sul trial che la barra rappresenta

Riprova / Continua

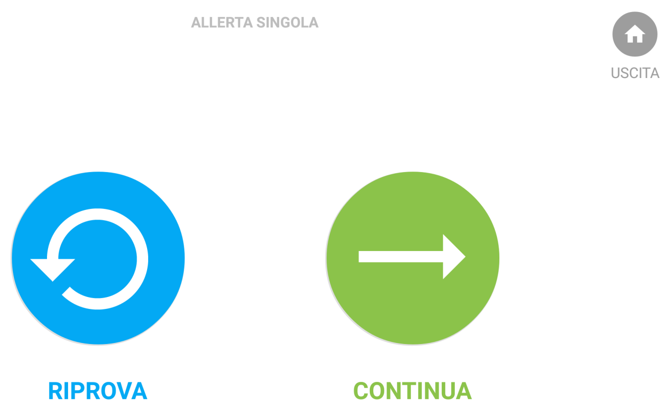

Figure 34. Un esempio di schermata riprova/continua alla fine di una sessione.

Subito dopo i grafici, viene presentata la schermata Riprova/Continua.

- Premendo "Riprova" si ricomincia la sessione appena giocata, senza mai salire di livello. Gli stimoli e la difficoltà rimangono pressoché uguali, a parte l'adattamento del gioco alle performance del paziente.
- Premendo "Continua" si prosegue con l'esercizio, eventualmente passando ai livelli successivi o **RETROCEDENDO** nel caso la performance sia stata particolarmente deficitaria.
- Con il tasto "casetta" in alto a destra, invece, si torna alla homepage.
